# Studying Respiratory Symptoms Related to Swimming Pools Attendance in Young Athletes: The SPHeRA Study

**DOI:** 10.3390/toxics10120759

**Published:** 2022-12-06

**Authors:** Matteo Zaccarin, Stefano Zanni, Francesca Gallè, Carmela Protano, Federica Valeriani, Giorgio Liguori, Vincenzo Romano Spica, Matteo Vitali

**Affiliations:** 1 Department of Public Health and Infectious Diseases, Sapienza University of Rome, 00185 Rome, Italy; 2 Department of Movement Sciences and Wellbeing, University of Naples “Parthenope”, 80133 Naples, Italy; 3 Department of Movement, Human, and Health Sciences, University of Rome “Foro Italico”, 00135 Rome, Italy

**Keywords:** youth, competitive swimmers, respiratory symptoms, chlorinated disinfection by-products, indoor air pollution

## Abstract

This study investigates the prevalence of respiratory symptoms and the training factors possibly associated with them in a sample of young Italian competitive swimmers. A questionnaire about training information and symptoms was administered to participants during the winter and summer 2021 training seasons. In total, 396 athletes took part in the study. In the winter training subgroup (*n* = 197), we found significant associations between increasing training hours per session and the presence of nasal congestion/rhinorrhoea (OR = 3.10; *p* = 0.039) and cough (OR = 3.48; *p* = 0.015). Total training hours per week were significantly associated with nasal congestion/rhinorrhoea (OR = 1.12; *p* = 0.010). In the summer group (*n* = 199), the same factors were not associated with respiratory symptoms. Having an allergy was significantly related to nasal congestion/rhinorrhea in both the logistic models (model 1 OR = 2.69, *p* = 0.013; model 2 OR = 2.70, *p* = 0.012), while having asthma significantly increased the risk of coughing (OR = 3.24, *p* = 0.033). The kind of environment (indoor or outdoor facilities) did not affect the studied symptoms either in summer or winter. Further investigations are needed to better understand the mechanisms involved in the development of respiratory symptoms in swimmers, particularly on how inflammation and remodelling develop and which environmental conditions can favour these processes.

## 1. Introduction

Swimming, and even more competitive swimming, certainly represents an adaptive challenge for the human body. Indeed, remaining in a horizontal position, immersed in a fluid other than the air in which our species evolved, certainly impacts positively on our physiology. Furthermore, the peculiar environment such as a swimming pool, with a high rate of pulmonary ventilation and fixed breathing patterns, dictated by a complex coordination of movements that ensure propulsion, may bring health benefits [1,2]. On the one hand, it can determine several positive effects on the respiratory health of swimmers. First, it is known that swimming seems to have a protective effect on asthmatic symptoms and it’s able to improve the respiratory fitness of patients and the peak of expiratory flow (PEF). This effect is probably due to the high relative humidity of the swimming pool’s environment [3]. Furthermore, swimming can also reduce the onset of symptoms and the frequency of asthma attacks, limiting the need for treatments [3,4,5]. Therefore, swimming has long been recommended as the most tolerable sport and recreational activity for asthmatics [6,7]. However, on the other hand, numerous studies evaluated the potential negative impact of swimming on practitioners. More specifically, respiratory symptoms such as coughing, dyspnoea, chest tightness, and throat or nasal problems have been observed to occur in competitive swimmers during training or close to the activity and to affect them more than other athletes or non-competitive swimmers [8,9]. One of the most discussed hypotheses suggests that the increased prevalence of these symptoms in competitive swimmers is due to the presence in water of disinfection by-products (DBPs) derived from chlorination [9,10,11,12,13,14,15], which is still the most widespread and economical method to control biological risk affecting the swimming pool water [16]. DBPs exposure to swimmers in swimming pools occurs primarily via the inhalation of volatile compounds or aerosol-containing DBPs and via the dermal route for those DBPs that are skin-permeable. DBPs present at higher levels in swimming pools are trihalomethanes (THMs) and haloacetic acids (HAAs), and THM concentrations in the pool environment were found at about 50 μg/L in water and 72 μg/m^3^ in the air [17,18].

Indeed, fewer rates of respiratory or irritative problems are reported in swimming facilities that use alternative processes of disinfection, such as UV rays, oxygen, copper, and ozone [19]. Based on data from the Italian National Institute of Statistics (ISTAT), continued sports practice in Italy increased from 15.9% of the population in 1995 to 24.5% in 2015, and water sports involved more than 4 million people. Swimming, specifically, is the most common sport among children aged 3 to 10 years [20], so exposure to DPBs derived from chlorination can occur very early. The aim of this study is to evaluate the prevalence of respiratory symptoms in young Italian competitive swimmers, considering the type of competitive season, and to assess for correlations between these symptoms and their swimming pool attendance.

## 2. Materials and Methods

### 2.1. Study Population

The cross-sectional study on Swimming Pools Health Related Aspects—SPHeRA—was carried out in 2021 on the whole population of Italian young competitive swimmers. All the swimmers met the criteria established by the Italian swimming federation (Federazione Italiana Nuoto, FIN) for participation at the Italian youth swimming championship, held at two different times during the competitive season. In particular, the first edition ended in April 2021 (winter/spring season) and the second one in August 2021 (summer season). These criteria, basing calculations on results from previous spring editions of the championship (from 2015 to 2019), selected an average population of 2015 athletes for each event, aged from 12 to 20 years old, of which 1099 was the average male number and 916 the female’s one. The choice to not focus on elite athletes has been guided by the aim of avoiding a potential selection bias. Indeed, respiratory symptoms can affect the quality of training and athletic performance of swimmers, potentially preventing them from reaching high levels of competition.

### 2.2. Study Design

Ideal sample size was estimated with an expected prevalence (*p*) of 0.5, a margin of error (ε) of 0.05 and a confidence level set at 95%. Thus, at least 385 people would have been necessary, while 351 would have been sufficient to adjust the estimate for the total number of athletes we expect, looking at results from previous editions of the championship. We tried to reach all the potentially eligible athletes on the Italian territory through an online self-administered questionnaire. To do that, every team with at least one swimmer registered for the championship has been contacted by e-mail and asked to invite their own eligible athletes to take part in our survey. The rationale behind the study, the instructions, and the web link to the questionnaire were included in the e-mail. December 2021 marked the closure and end of data collection. The study was carried out according to the principles of the Declaration of Helsinki. Ethical approval was obtained from the Research Committee of the University of Rome “Foro Italico” (approval n CAR 92/2021).

### 2.3. Questionnaire

The questionnaire was created using other previously validated questionnaires, adapting questions to our specific population and intent [21,22]. The first part of the questionnaire was aimed at collecting general information about each athlete, such as sex, age, years of practice, the duration of their training, and the number of training sessions per week. The latter two pieces of information allowed us to also get the total amount of weekly swimming hours. The second part was focused on the presence of respiratory diseases and symptoms such as nasal congestion/rhinorrhoea, sneezing, coughing, itchy throat, wheezing, chest tightness, and breathing difficulty, occurring during or immediately after the training sessions; the intensity of these symptoms, if any, and their possible impact on sleep quality were also investigated. We asked participants to report only symptoms not related to infectious diseases suffered. The period to which the questions referred roughly included the three months prior to the competition. The questionnaire underwent a validation process. In particular, it was subjected to expert and non-expert review and a pilot test was carried out on a subset of eligible swimmers (*n* = 86) to evaluate understandability, consistency, validity, and possible floor or ceiling effects inside the answer range. In these phases, some questions considered not relevant were removed and some other questions were modified according to the suggestions of the pilot participants. Cronbach’s alpha was calculated for assessing the reliability of test items, and it resulted in >0.80 for all the examined areas.

### 2.4. Statistical Analysis

Data analysis was performed on the statistical software STATA^®^ (STATA 17.0, StataCorp LLC, College Station, TX, USA). At first, descriptive analysis was carried out and arithmetic mean, median, and standard deviation (SD) were calculated for each continuous variable. We used the Shapiro-Wilk test to assess the normality of data. Dichotomous variables were compared by chi-square test (χ^2^); the comparison between dichotomous and continuous variables was performed using the non-parametric Wilcoxon rank sum test. We consider the dichotomous variables associated with the presence or absence of respiratory symptoms as outcomes: nasal congestion/rhinorrhea, sneezing, sore throat, coughing, wheezing, chest tightness, and difficulty breathing. We applied the simple logistic regression model for univariable analysis, and then predictor variables found to be significant in the univariable models were fitted into multiple logistic regression models. In addition, we included also “having asthma”, “having allergy”, and the kind of training environment (indoor or outdoor facilities) as independent variables of the regression models. Sex and age variables were included in these models as covariates. Each model was stratified according to the training season, for those outcomes that had significant seasonal differences. Significance was considered at *p* < 0.05. We included in our analysis a variable related to the hours of training per week as an indicator of longer exposure than the training hours in a single session, obtained by multiplying hours of training in a single session by the number of training sessions per week.

## 3. Results

The main characteristics of the study population obtained through a questionnaire and the results of descriptive analysis, considering all the participants and the subgroups according to the season, were shown in Table 1.

Overall, a total of 396 athletes completed the questionnaire in every section and gave their consent to process data. 51.0% of the participants were female with a mean age equal to 16 years ± 2. Regarding the winter/spring swimming season, 197 athletes answered the questionnaire, with a participation rate of 10% of the expected population, while in the summer, 199 swimmers replied to the survey (participation rate of 10.3%). The two groups differed in the distance swum per session and training practiced indoors, with both being higher in the winter/spring season. Around 10% of swimmers stated that they suffered from asthma, ascertained by a doctor’s diagnosis, and about half of these subjects required pharmacological treatment. The prevalence of studied swimmers having a diagnosed allergic disease was 21.2%, and 60% of allergic participants used medication regularly or as needed. The most frequent allergens were dust mites and pollen, both of which were reported by more than half of the allergy sufferers. Almost all the swimmers (97.2%) stated that they had never smoked cigarettes, while 1.0% and 0.8% were respectively ex-smokers and current smokers, with an average consumption of twenty cigarettes per week. Two-thirds of the swimmers who reported having nasal congestion and/or rhinorrhea during and/or after the training experienced the symptom at almost every training session for at least once a week, and the other third less than once a week. These symptoms generally did not affect training (88.0% of cases). The quality of sleep was completely compromised for 7.5% of these swimmers, while almost half of them experience a worsening and/or showed no change in comparison to nights without the symptom (44.6% and 47.8% respectively). Sneezing was the most frequently reported symptom, and half of the swimmers who experienced it associated it with almost every swimming session or at least every week of training. The sensations of tingling and/or itching in the throat did not affect the activity in the pool in more than half of the population; 26.3% reported a compromise in the quality of the training session, and 8.1% needed to stop swimming. The symptom of coughing is experienced almost always or at least every week in more than half of the athletes who cough. It was so intense that it compromised the activity in the water for 45.5% of them, and 10% were forced to stop training. In 9% of the cases, coughing totally compromised sleep, and in 25% it negatively affected sleep quality, while for 65.3% it had no impact on it. Regarding the presence of wheezing, 10% of the subjects reporting it experienced the symptom almost every time they trained, 40% at least once a week, and 50% less than once a week. This symptom did not affect training in 62.7% of cases; in the remaining part, it compromised the workout quality, with 12% of athletes forced to interrupt their activity. Talking about the feeling of chest tightness during and/or after training, 13.3% of those who experienced it stated that they feel it almost every time they train, 33.3% at least once a week and 53.3% less than once a week. The intensity of the symptom in more than half of them lowered the quality of their daily performance in the water, and 8% of those experiencing it were unable to continue their activity. In the part of swimmers who faced difficulty in breathing, almost 60% had difficulty every training session or at least once a week. The intensity of this symptom did not allow the continuation of the swimming activity for 7.1% of these athletes; 54.5% saw the quality of their training compromised; 38.4% did not see any influence on their training session. In the univariable analyses (Table 1), significantly higher proportions were found for congestion/rhinorrhea (*p* = 0.021), coughing (*p* < 0.001), chest tightness (*p* = 0.006) and breathing difficulty (*p* = 0.024) in winter training session respect to summer one. 

The results of simple logistic regression analyses performed to evaluate the effect of some predictors on respiratory symptoms for all the participants are presented in Table S1. As reported in Table S1, significant differences were found for congestion/rhinorrhea, coughing, and breathing difficulty in relation to the hours of training per session and for congestion/rhinorrhea and breathing difficulty in relation to hours of training per week.

The results of simple logistic regression analyses performed to evaluate the effect of some predictors on respiratory symptoms for the subgroups divided according to the season are shown in Table 2.

Considering the subgroups stratified according to the season (Table 2), the outcome of congestion/rhinorrhea was associated with a significant OR increase related to the number of training sessions per week, the hours of training per session and the hours of training per week. For the coughing outcome, we found a significant increase in OR in relation to hours of training per session and distance swum per session. The same models in the summer group were not significant for any variables. For chest tightness and breathing difficulty, no significant associations were found in winter or in summer (data not shown). 

Those variables which showed a relationship with symptoms in the simple logistic model were then included as independent variables in the three multivariable logistic models, together with sex and age as covariates. Nasal congestion/rhinorrhea was used as a dependent variable in the first and the second model, while coughing was used as a dependent variable in the third model. Hours of training per session and hours of training per week were not studied in the same regression analysis due to possible collinearity in the fitting model. The results of multiple logistic regression models are shown in Table 3.

In all the models, age and sex were not associated with the studied dependent variables. In the group of athletes who trained in winter, the multivariable analysis revealed significant associations between nasal congestion/rhinorrhea and training hours per session (OR = 3.10, *p* = 0.039) in the first model and hours of training per week in the second model (OR = 1.12, *p* = 0.010); coughing was found to be related with the number of hours per session (OR = 3.48, *p* = 0.015). In the summer, none of the factors related to the duration of training were associated with the symptoms reported. Having an allergy was significantly related to nasal congestion/rhinorrhea in both the logistic models (model 1 OR = 2.69, *p* = 0.013; model 2 OR = 2.70, *p* = 0.012), while having asthma significantly increased the risk of coughing (OR = 3.24, *p* = 0.033). The kind of environment (indoor or outdoor facilities) did not affect the studied symptoms either in summer or winter.

## 4. Discussion

This study was aimed at exploring the prevalence of respiratory symptoms in young Italian competitive swimmers. Numerous studies have reported a high incidence of respiratory symptoms in competitive swimmers [8,23,24]. In particular, in the study performed by Päivinen et al. [8], respiratory symptoms were reported by 18% of the studied swimmers. A higher proportion was found by Stadelmann et al. [23], which recovered respiratory symptoms in 83% of the sample. Furthermore, Bougault et al. [22] found rhinitis symptoms in 74% of swimmers during an intense training period.

These athletes, indeed, train in a unique environment compared with other elite athletes, characterized by increased temperature and humidity and containing a great number of chemicals [25,26]. In particular, early and chronic exposure, typical of young competitive swimmers, appears to have a promoting effect on airway inflammation, hyperreactivity, and on the process of allergic sensitization [25]. However, conflicting results and bias due to study design, diverse environmental conditions, the number of subjects who swam in the same pool, different times and ways of exposure, and seasons emerged from previous studies performed both in the general population and in competitive swimmers [18,27,28,29,30]. 

The COVID-19 pandemic has represented an opportunity for studying the association between swimming pool attendance and respiratory system syndromes under controlled conditions. Indeed, due to social distancing, hand hygiene and masking measures, a lot of scientific studies have reported a reduction in seasonal viral infections and associated respiratory symptoms and thus fewer confounding factors [31,32,33,34]. In this context, this study attempted to assess, in young athletes involved in competitive swimming by means of a survey, respiratory symptoms not related with infectious diseases, but most likely related to the exposure to the swimming pool environment. The results of several studies showed that competitive swimmers have a relevant prevalence of symptoms (nasal congestion/rhinorrhea and coughing) associated with attendance at swimming pools [8,9,22,23]. In accordance with the findings of these studies, our sample showed a prevalence of nasal congestion/rhinorrhea and coughing equal to 47% and 42.2%, respectively, and significantly higher in winter than in the summer season. These findings support the hypothesis of an irritative stimulus from the environment in which the swimming activities take place, and that it may be related to the presence in the air of chlorination disinfection by-products. It is arguable that in winter, when training took place mainly in closed and poorly ventilated environments, the increase in concentrations of airborne by-products would result in higher exposure of athletes than in summer, when training is carried out outdoors or in partially closed environments. In fact, our results show that training indoors is more frequent during the winter/spring season. However, no associations or negative associations were found in the regression analyses between respiratory symptoms and the type of facility attended. Instead, besides asthma and allergies, the variables related to training volume were found to be significantly related with the outcomes. When considering the season subgroups, these relationships were confirmed only in the winter/spring group. 

Respiratory symptoms generally manifest themselves after training [35]. Indeed, during and after exercise, physiological modifications take place due to adrenergic and parasympathetic effects, and after the exercise, bronchoconstriction and exercise-related bronchospasm arise [36,37]. As previously reported, the intensity of exercise, together with environmental factors, such as temperature, quality of air, and humidity, can lead to bronchoconstriction [38]. Our results are in line with this evidence since they suggest an interaction between environmental conditions and training factors in determining respiratory symptomatology in swimmers.

Other considerations must be made about sleep. In athletes, sleep is needed for recovery [39], and observing sleep hygiene is a key point for the performance of elite athletes [40]. In our sample, 7.5% of participants reported that the quality of sleep was completely compromised. However, almost half of them experience no changes in comparison to nights without the symptoms, underlining that several other factors can affect sleep, as reported in the literature [41,42,43].

This study presents several limitations. First, this is a cross-sectional study and, consequently, general statements on the causal or temporal relationship cannot be made, and internal validity can be negatively affected by confounding factors. However, this kind of study is inexpensive, easy, and fast to conduct; furthermore, they are the best way to measure the prevalence of health outcomes and are useful to hypothesize determinants of health, study multiple outcomes and exposures, and describe the features of a population [44]. Thus, we considered this type of study design as the most appropriate to achieve our objective by reaching the whole Italian young competitive swimmers population. Second, athletes can suffer stress from other sources, such as academic, social, lifestyle, and athlete coach-relationship, and some of these factors could have also influenced the respiratory syndromes. Third, data were self-reported, and the monitoring period (two seasons of one year) was too short and does not reflect the long-term exposure of the swimmers. In addition, no data on chemicals used in the water or on environmental conditions of swimming facilities such as air quality or the number of individuals that swam in the same pool to which refer individual information were collected. Lastly, the age range of the participants may have affected the results as biological maturational age can influence the response to chemical exposure.

However, this study has some strengths related with the investigation period and the characteristics of the sample. Indeed, the COVID-19 pandemic allowed the reduction in the possibility of respiratory symptoms determined by seasonal viral infections, which were extremely low during the pandemic. Furthermore, in the study period, the levels of air pollution, another known risk factor for respiratory symptoms, significantly decreased in Italy [45,46]. Thus, negative short-term effects on the respiratory system deriving from exposure to air pollution have been considerably mitigated. In addition, we found a very low frequency of smokers, as resulted also in previous studies performed by other researchers [47], and of asthmatics among the participants. Having a cohort without smokers and asthmatics is of great relevance when exploring respiratory symptoms because it allows for controlling the role of tobacco smoke or of asthma as important confounding factors. In fact, smoking or asthma may determine some negative effects on respiratory function and other respiratory symptoms, similar to those reported in swimmers or other subjects exposed to airborne pollutants [8,47,48]. However, in order to evaluate the contribution of allergy and asthma to respiratory symptoms, we added these two variables in the logistic models and, in summer, having allergy or asthma were independent contributors, respectively, to nasal congestion/rhinorrhea and coughing.

Associating self-reported symptomatology with exposure data and water/air sampling, aimed particularly at assessing parameters such as the concentration of free and combined chlorine, THM and other DBPs, could strengthen the hypothesized correlation between swimming pool attendance and respiratory symptoms. Further research should follow this direction.

## 5. Conclusions

The results of the present study evidenced a prevalence of respiratory symptoms during or immediately after training ranging from 16.9% to 47% in the sample examined. Significantly higher proportions in the winter training sessions with respect to summer ones were found for some of these respiratory symptoms, such as congestion/rhinorrhea, coughing, chest tightness, and breathing difficulty. Furthermore, during the winter season, training hours per session and per week were significantly associated with different respiratory symptoms among competitive swimmers, while during the summer season the same associations were not recovered. The type of facility attended was found not to be associated with the outcome. Further investigations are needed to better understand the mechanisms involved in the development of respiratory symptoms in swimmers, particularly on how inflammation and remodelling develop, and which training or environmental conditions can favour these processes. These aspects could help to better address the environmental parameters monitoring in order to prevent respiratory symptoms in swimmers.

## Figures and Tables

**Table 1:** Main characteristics of the study population and results of descriptive analysis, considering all the participants and the subgroups according to the season.

Variable	Total N = 396	Winter/Spring Season N = 197	Summer Season N = 199	*p*-Value
Female athletes *n* (%)	202 (51.0)	93 (47.2)	109 (54.8)	0.132
Male athletes *n* (%)	194 (49.0)	104 (52.8)	90 (45.2)	
Age (years) arithmetic mean ± SD	16 ± 2	16 ± 6	16 ± 5	0.775
Years of practice arithmetic mean ± SD	8 ± 3	8 ± 4	8 ± 4	0.460
Training sessions per week arithmetic mean ± SD	6 ± 2	6 ± 1	6 ± 4	0.050
Hours of training per session arithmetic mean ± SD	2.0 ± 0.3	2.0 ± 0.3	2.0 ± 0.3	0.100
Distance swum per session (Km) arithmetic mean ± SD	5.8 ± 2	6.1 ± 2	5.6 ± 3	0.001
Hours of training per week arithmetic mean ± SD	12 ± 5	12 ± 4	13 ± 5	0.356
Indoor training *n* (%)	295 (74.5)	195 (99.0)	100 (50.3)	<0.001
Diagnosed asthma *n* (%)	38 (9.6)	21 (10.7)	17 (8.5)	0.474
Diagnosed allergic diseases *n* (%)	84 (21.2)	48 (24.4)	36 (18.1)	0.127
Respiratory symptoms experienced during or after training:				
Nasal congestion/rhinorrhea *n* (%)	186 (47.0)	104 (52.8)	82 (41.2)	0.021
Sneezing *n* (%)	247 (62.4)	130 (66.0)	117 (58.8)	0.139
Coughing *n* (%)	167 (42.2)	101 (51.3)	66 (33.2)	<0.001
Itchy throat *n* (%)	99 (25.0)	56 (28.4)	43 (21.6)	0.117
Wheezing *n* (%)	67 (16.9)	37 (18.8)	30 (15.1)	0.325
Chest tightness *n* (%)	75 (18.9)	48 (24.4)	27 (13.6)	0.006
Breathing difficulty *n* (%)	99 (25.0)	59 (29.9)	40 (20.1)	0.024

**Table 2:** Results of simple logistic regression analysis in the participants grouped according to the season.

Outcomes	Predictors	Winter		Summer	
		OR (95% CI)	*p*-Value	OR (95% CI)	*p*-Value
Nasal congestion/rhinorrhea	Age	1.03 (0.95–1.12)	0.432	0.95 (0.85–1.06)	0.378
	Sex	1.38 (0.78–2.42)	0.265	1.67 (0.94–2.97)	0.079
	Years of practice	1.01 (0.93–1.09)	0.827	0.99 (0.90–1.09)	0.850
	Training sessions per week	1.27 (1.01–1.61)	0.040	0.95 (0.82–1.09)	0.474
	Hours of training per session	3.53 (1.33–9.37)	0.011	0.96 (0.38–2.40)	0.923
	Distance swum per session (Km)	1.00 (0.83–1.20)	0.992	1.11 (0.91–1.35)	0.284
	Hours of training per week	1.11 (1.02–1.21)	0.014	0.98 (0.93–1.04)	0.588
	Indoor training	0.89 (0.05–14.48)	0.937	0.94 (0.53–1.65)	0.819
	Diagnosed asthma	0.98 (0.40–2.43)	0.968	1.68 (0.62–4.55)	0.308
	Diagnosed allergic diseases	1.51 (0.78–2.92)	0.225	2.70 (1.28–5.66)	0.009
Coughing	Age	0.92 (0.85–1.01)	0.085	0.96 (0.86–1.08)	0.484
	Sex	0.80 (0.46–1.41)	0.444	1.72 (0.94–3.16)	0.078
	Years of practice	0.97 (0.89–1.05)	0.438	0.97 (0.88–1.07)	0.562
	Training sessions per week	0.96 (0.81–1.23)	0.969	0.98 (0.85–1.14)	0.806
	Hours of training per session	3.63 (1.36–9.66)	0.010	1.08 (0.41–2.83)	0.870
	Distance swum per session (Km)	1.23 (1.02–1.49)	0.034	1.02 (0.84–1.25)	0.807
	Hours of training per week	1.04 (0.96–1.12)	0.313	1.00 (0.94–1.07)	0.882
	Indoor training	0.95 (0.06–15.40)	0.971	0.64 (0.35–1.17)	0.147
	Diagnosed asthma	2.05 (0.79–5.31)	0.141	3.21 (1.16–8.88)	0.024
	Diagnosed allergic diseases	1.46 (0.75–2.81)	0.261	1.81 (0.86–3.78)	0.115

**Table 3:** Results of multiple logistic regression analysis.

Outcomes	Predictors	Winter		Summer	
		OR (95% CI)	*p*-Value	OR (95% CI)	*p*-Value
		Logistic model 1: pseudo R^2^ = 0.0488		Logistic model 1: pseudo R^2^ = 0.0421	
Nasal congestion/rhinorrhea	Age	1.04 (0.95–1.14)	0.372	0.98 (0.87–1.10)	0.724
	Sex	1.42 (0.79–2.58)	0.239	1.74 (0.91–3.32)	0.091
	Training sessions per week	1.18 (0.92–1.52)	0.199	0.99 (0.85–1.16)	0.945
	Hours of training per session	3.10 (1.05–9.08)	0.039	0.95 (0.35–2.59)	0.925
	Asthma	0.78 (0.26–2.30)	0.650	1.35 (0.46–3.99)	0.582
	Allergy	1.68 (0.76–3.73)	0.204	2.69 (1.23–5.88)	0.013
	Indoor/outdoor facilities	0.47 (0.02–11.44)	0.645	0.91 (0.50–1.65)	0.753
		Logistic model 2: pseudo R^2^ = 0.0410		Logistic model 2: pseudo R^2^ = 0.0420	
Nasal congestion/rhinorrhea	Age	1.02 (0.94–1.12)	0.531	0.98 (0.87–1.10)	0.725
	Sex	1.44 (0.80–2.61)	0.212	1.73 (0.91–3.30)	0.090
	Hours of training per week	1.12 (1.03–1.22)	0.010	1.00 (0.94–1.06)	0.920
	Asthma	0.79 (0.27–2.30)	0.202	1.35 (0.46–3.93)	0.585
	Allergy	1.67 (0.76–3.66)	0.663	2.70 (1.24–5.88)	0.012
	Indoor/outdoor facilities	0.37 (0.013–10.72)	0.562	0.91 (0.50–1.65)	0.756
		Logistic model 3: pseudo R^2^ = 0.0487		Logistic model 3: pseudo R^2^ = 0.0488	
Coughing	Age	0.92 (0.83–1.02)	0.123	1.00 (0.88–1.13)	0.994
	Sex	0.76 (0.42–1.39)	0.375	2.04 (0.98–4.07)	0.052
	Hours of training per session	3.48 (1.28–9.50)	0.015	0.89 (0.33–2.42)	0.825
	Asthma	1.58 (0.53–4.76)	0.413	3.24 (1.09–9.59)	0.033
	Allergy	1.39 (0.64–3.03)	0.406	1.53 (0.70–3.36)	0.289
	Indoor/outdoor facilities	0.80 (0.05–13.34)	0.380	0.57 (0.30–1.06)	0.080

## Data Availability

The datasets generated during and/or analysed during the current study are available from the corresponding author on reasonable request.

## Supplementary Materials

The following supporting information can be downloaded at: https://www.mdpi.com/article/10.3390/toxics10120759/s1, Table S1: Results of simple logistic regression analysis in all the participants.
