# Supplementary material for: Studying Respiratory Symptoms Related to Swimming Pools Attendance in Young Athletes: The SPHeRA Study
Source: Toxics. 2022 Dec 6;10(12):759. doi: 10.3390/toxics10120759 (PMC9784475; doi:10.3390/toxics10120759)
Supplement: Supplementary file 1 [file toxics-10-00759-s001.zip › toxics-1996367-supplementary.pdf]

Table S1. Results of simple logistic regression analysis in all the participants.

Outcomes	Predictors	TOTAL	
		OR (95%CI)	*p-value*
**Nasal congestion/rhinorrhea**	Age	1.01 (0.95-1.07)	0.816
	Sex	1.45 (0.97-2.15)	0.067
	Years of practice	1.00 (0.94-1.06)	0.977
	Training sessions per **week**	1.02 (0.91-1.15)	0.727
	Hours of training per **session**	1.90 (0.99-3.65)	0.054
	Distance swum per **session** (Km)	1.07 (0.94-1.22)	0.285
	Hours of training per **week**	1.02 (0.98-1.07)	0.296
	Indoor training	0.71 (0.45-1.12)	0.138
	Diagnosed asthma	1.28 (0.66-2.51)	0.463
	Diagnosed allergic diseases	2.03 (1.24-3.31)	0.005
**Coughing**	Age	0.94 (0.88-1.01)	0.098
	Sex	1.08 (0.72-1.61)	0.712
	Years of practice	0.97 (0.91-1.03)	0.287
	Training sessions per **week**	0.96 (0.86-1.09)	0.558
	Hours of training per **session**	2.14 (1.10-4.17)	0.024
	Distance swum per **session** (Km)	1.17 (1.02-1.34)	0.025
	Hours of training per **week**	1.01 (0.97-1.06)	0.580
	Indoor training	0.46 (0.28-0.75)	0.002
	Diagnosed asthma	2.58 (1.29-5.15)	0.007
	Diagnosed allergic diseases	1.69 (1.04-2.74)	0.034
**Breathing difficulty**	Age	1.01 (0.94-1.08)	0.755
	Sex	1.27 (0.81-2.01)	0.297
	Years of practice	1.06 (0.99-1.14)	0.081
	Training sessions per **week**	1.06 (0.93-1.21)	0.405
	Hours of training per **session**	2.34 (1.12-4.91)	0.024
	Distance swum per **session** (Km)	1.09 (0.94-1.27)	0.260

	Hours of training per **week**	1.04 (0.99-1.10)	0.088
	Indoor training	0.67 (0.39-1.17)	0.164
	Diagnosed asthma	2.72 (1.37-5.41)	0.004
	Diagnosed allergic diseases	2.96 (1.77-4.95)	<0.001
